# Cooperation or Competition of the Two Hemispheres in Processing Characters Presented at Vertical Midline

**DOI:** 10.1371/journal.pone.0057421

**Published:** 2013-02-22

**Authors:** Rolf Verleger, Marie Dittmer, Kamila Śmigasiewicz

**Affiliations:** Department of Neurology, University of Lübeck, Lübeck, Germany; University Medical Center Groningen UMCG, The Netherlands

## Abstract

Little is known about how the hemispheres interact in processing of stimuli presented at vertical midline. Processing might be mutually independent or cooperative. Here we measured target identification and visually evoked EEG potentials while stimulus streams containing two targets, T1 and T2, were either presented at vertical midline above and below fixation, or laterally, left and right. With left and right streams, potentials evoked by filler stimuli and by T2 were earlier at the right than the left visual cortex, and T2 was better identified left than right, confirming earlier results and suggesting better capabilities of the right hemisphere in this task. With streams above and below fixation, EEG potentials evoked by filler stimuli and by T2 were likewise earlier at the right than the left hemisphere, and T2 was generally identified as well as, but not better than left T2, in one target constellation even worse (T2 in lower stream preceded by T1 in upper stream). These results suggest right-hemisphere preference for this task even with stimuli at vertical midline, and no added value through hemispheric cooperation. Lacking asymmetry for T1 amidst asymmetries for filler stimuli and for T2 might indicate alternating access of the hemispheres to midline stimuli as one means of hemispheric division of labor.

## Introduction

Visual stimuli presented in the left or right visual field activate the contralateral primary visual cortex [1], [2]. Since there is no middle visual cortex, stimuli presented at vertical midline likewise activate the left or right primary visual cortex [1], [3] or both [4], [5]. Having arrived in one visual cortex, stimulus-induced activation may pass to the other hemisphere via the corpus callosum or subcortical pathways [6].

The two visual cortices differ in their preferences of processing, i.e., either hemisphere processes certain features of stimuli better than the other [7], [8] (see supplementary video to [9] for a striking example of spatial processing in a patient with damaged corpus callosum). A marker of preferential processing is the early activation of visual areas downstream from primary visual cortex, as reflected by the N1 component of the event-related EEG potential (ERP) around 170 ms after stimulus onset. For example, N1 is larger at left than right posterior recording sites for centrally presented words, suggesting preferential processing of words in the left visual cortex and, similarly, N1 is larger for faces than for cars or words at right sites, suggesting better processing of faces than of the other stimuli in the right visual cortex [10]. Thus, one hemisphere may have a head start, by being contralateral to lateral stimuli or by its preferences of processing.

With the modules in either hemisphere being activated, these two systems may interact in different ways when processing vertical-midline stimuli. One possibility is *cooperation*: By exchanging and adding to each other's information, the two hemispheres will process midline stimuli better than either one could do alone. Another possibility is *racing*: Each hemisphere will process the information it has acquired (directly or via interhemispheric transmission) and the faster result will determine the output of the entire visual system. In this case, midline stimuli will be processed as well as the better hemisphere would do alone, but not better (i.e., without "redundancy gain", cf. [11]). At the end of such racing, the output of the other hemisphere would possibly need to be inhibited, to achieve unambiguity. This notion leads to a third possibility, *mutual inhibition* [12]: The one hemisphere that has a head start will inhibit the other hemisphere early in processing already. In this paper, we will not distinguish between racing and mutual inhibition but between the former two alternatives: racing (implying mutual inhibition) and cooperation (implying mutual boosting).

Some studies have used bilateral rapid serial visual presentation of letters and digits for assessing the abilities of the two hemispheres in detecting and identifying relevant target stimuli in rapidly changing environments [13]-[18]. Two targets, T1 and T2, had to be identified in each trial. Either target could be on the left or on the right. While T1 was identified equally well on both sides, left T2 was better identified than right T2, particularly though not only when T1–T2 lag was short and when T1 and T2 were in different streams. This asymmetry suggests better processing of rapidly presented information in the right hemisphere.

The present study tested whether such better processing of left than right lateral rapidly presented targets translates to preferential processing of target streams presented at vertical midline in the right hemisphere, just like words presented at midline are preferentially processed in the left hemisphere, and faces in the right one. To have conditions comparable to the two streams left and right, vertical-midline stimuli were not presented at fixation but in two streams, above and below fixation.

The following parameters had been found to indicate the right-hemisphere advantage in the above-mentioned studies and, therefore, will here be measured as indicators of asymmetries in processing midline stimuli.

T2 identification: T2 at vertical midline may be identified either as well as on the left or as poorly as on the right, or even better than on the left. The former alternative would speak for right-hemispheric processing and, at the same time, for the racing model, the middle for left-hemispheric processing and the racing model, allowing for the possibility that the less suitable ("non-preferential") left hemisphere may nevertheless get faster access to T2 and determine the output, and the latter alternative would speak for hemispheric cooperation.

VEPs (visually evoked potentials): VEPs evoked by the constant bilateral stream of filler stimuli in this task were leading by a few milliseconds in right- compared to left-hemisphere recordings [18]. If also obtained for midline stimuli, this lag between right and left will speak for a constant setting of preferential processing in the right hemisphere.

T1-evoked PCN (posterior contralateral negativity): Being a relevant event, laterally presented T1 had evoked PCN in previous studies [15], [18] peaking around 200 ms after T1 onset. PCN is considered a marker of selective processing [19]-[21]. Thus, any PCN-like asymmetry evoked by midline-T1 may indicate preferential processing in the hemisphere that will display more negativity. Larger PCN amplitudes were obtained at the left hemisphere (for right T1) than at the right (for left T1) by us in unpublished data but not in [18]. Thus, tentatively, larger negativity at the left hemisphere is predicted for T1 presented at midline which would suggest preferential processing of T1 in the left hemisphere. (This PCN was termed "N2pc" in our previous dual-stream studies [15], [18] using the label introduced by Luck et al. [19]. However, since N2pc means "N2 posterior contralateral" and T1- and T2-evoked PCN actually overlapped with the posterior N1 (peaking at about 190 ms) rather than with some later N2, we will use the more neutral term PCN [22], [23].)

T2-evoked PCN: Likewise being a relevant event, laterally presented T2 also evokes its PCN [15], [18], [24]. Again, any PCN-like asymmetry evoked by midline-T2 may indicate preferential processing in the hemisphere that will display more negativity. We assume that negativity will be larger at the right than the left hemisphere, in accordance with right-hemispheric preference for processing midline T2.

To summarize, the listed ERP indicators are expected to provide evidence for right-hemispheric preference in processing of filler stimuli and of T2, and possibly for left-hemispheric preference in processing of T1. Clues about cooperation vs. race among the hemispheres will be obtained by comparing the identification rates of vertical-midline to lateral stimuli.

## Methods

### Participants

The project was approved by the ethical committee of the University of Lübeck (ref. no. 11–198). Twenty-three students (11 male) participated, aged 20 to 27 years (mean = 23, SD = 1.9). Informed written consent was obtained and 7 € per hour were paid. All participants reported normal or corrected-to-normal vision, normal color vision, and no history of neurological disorders. Most of them were right-handed and two were ambidextrous, with scores of 83 ±22 (range 25–100) in the Edinburgh Handedness Inventory. Five participants were rejected from analysis due to systematic eye movements toward the target (s. below, EEG pre-processing), one because of high error rates in identifying T1 (54% correct, the average from the other 20 participants being 81% ±12%) and another one because of high error rates in identifying T2 (42% correct, the average from the other 20 participants being 75% ±16%). Thus, 16 participants remained for analysis (9 male).

### Stimuli and Apparatus

**Figure 1:**
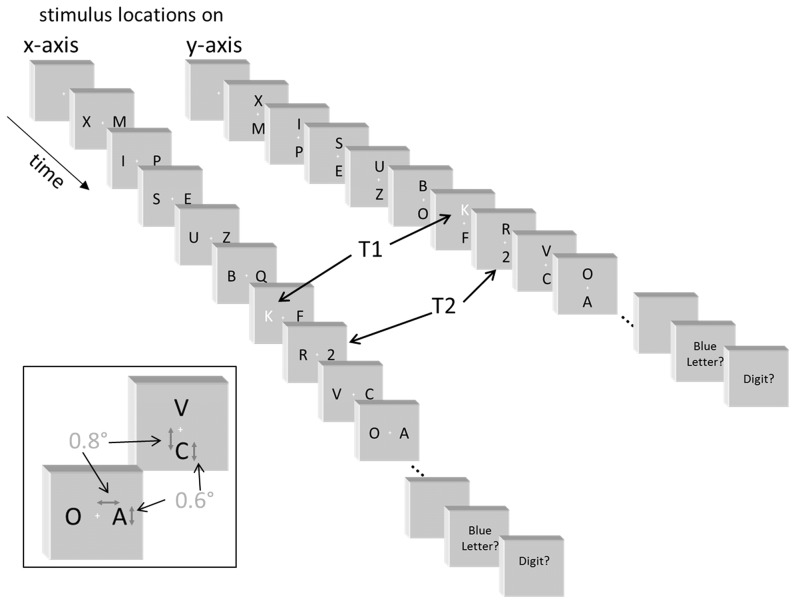
Sequence of events in a trial of x-axis and y-axis blocks. See Methods for details. Blue color of the 1^st^ target is here replaced by white color. In this example, T1 and T2 are in different streams with a lag of 1 frame. The inset in the lower left corner shows the size relationships: Letters were 0.6° high, with 0.8° distance of their midpoints from screen center.

The task is illustrated in Figure 1. Two simultaneous sequences of black (1 cd/m^2^) capital letters of the Latin alphabet were rapidly (7.7/s) presented on the white background (120 cd/m^2^) of a 17’’ screen driven with 85 Hz at about 1.1 m from participants' face. (Luminance indications as obtained from an LXcan luminance meter, Scanditronix Wellhöfer, Germany). Letter font was Helvetica 35, thus letters were 11 mm high (0.6° visual angle). Their midpoints were 16 mm off screen center (0.8°), implying distances of their inner edges from screen center of 10 mm (0.5°) for stimuli above and below center and of around 11 mm (varying between letters) for left and right stimuli. Fixation was marked by a small red cross (0.1°×0.1°) at screen center. In each trial, two targets had to be identified. The first target (T1) was a blue letter (24 cd/m^2^; D, F, G, J, K, or L; selected because of their neighboring positions on the keyboard). The second target (T2) was a black digit (1, 2, 3, 4, 5, or 6). The filler set consisted of all letters in black. Presentation® software, version 14.5, was used for experimental control (Neurobehavioral Systems Inc.). Fixation was controlled by means of an infra-red remote eye tracker (Eyegaze Analysis System, Interactive Minds, Dresden, Germany) which communicated on-line with the Presentation program.

In one block of trials, the two stimulus streams were left and right from fixation ("x-axis" block), like in previous studies. In the other, "y-axis", block, the two stimulus streams were above and below fixation.

### Procedure

Participants were seated in a comfortable armchair in a dimly lit laboratory room in front of the computer screen. The EEG recording was prepared, as detailed below. Then gaze directions were mapped to screen coordinates for use of the eye-tracker, using the calibration routine provided by the eye-tracker manufacturer where participants had to fixate nine positions on the screen in fixed sequence. Afterwards, the experimental task was started. Each trial began with onset of the fixation cross, followed after 1.8 s by the series of letters. Each pair of letters was presented for 130 ms (precisely 129.4 ms), immediately followed by the next frame. Five, seven, or nine letter pairs preceded T1 (to make precise temporal expectancies difficult [25]). T1 and T2 were presented in either stream with equal probability independently from each other. T2 followed T1 with lags of 130 ms (Lag 1) or 520 ms (Lag 4). Five letter-pairs followed T2. Therefore trial length varied between 12 frames (T1 at 6^th^ position and T1–T2 lag of 1) and 19 frames (T1 at 10^th^ position and T1–T2 lag of 4). Then the fixation cross went off and prompts appeared on the screen to enter identities of T1 and T2, first the T1 letter on the middle row of a standard keyboard and then the T2 digit on its number pad. Some response had to be given even if the answer was not known. Then the next trial started. In each trial, filler stimuli were randomly selected with replacement from the letter set (but immediate repetitions and identical stimuli in a frame were excluded), and T1 and T2 were randomly selected from the target sets. If the eye-tracker registered a deviation of more than 0.4° from fixation at trial onset, a big red exclamation mark (font 50) was presented at center for 2 s, leading participant's gaze back to fixation and restarting the trial after another 0.5 s.

These experimental settings resulted in three relevant dimensions: T1 location (left or right in the x-axis block; up or down in the y-axis block), lag between T1 and T2 (1 or 4), and T2 location (same as T1 or opposite to T1), resulting in eight equiprobable combinations that were replicated 45 times in random sequence, making up a block of 360 trials. The two stimulus streams were horizontally or vertically aligned to screen center, in other words they flanked fixation either on the x-axis or on the y-axis. The session consisted of one x-axis and one y-axis block, with their order balanced between the 16 participants of the final sample. Before the task proper, some trials were presented in slow motion for practice, with 500 ms presentation time per frame.

### EEG recording and pre-processing

EEG was recorded with Ag/AgCl electrodes (Easycap, www.easycap.de) from 60 scalp sites, including 8 midline positions from AFz to Oz and 26 pairs of symmetric left and right sites. On-line reference was Fz, data were off-line re-referenced to the nose-tip. The ground electrode was placed at Fpz. For artifact control, vertical EOG was recorded from above vs. below the right eye, horizontal EOG (hEOG) from positions next to the outer rims of the eyes. Data were amplified from DC to 250 Hz by a BrainAmp MR plus and stored at 500 Hz per channel.

Further processing was done with Brain-Vision Analyzer software (version 2.01). After re-referencing and low-pass filtering at 20 Hz, data from each trial were split into appropriate segments for analysis of filler-evoked VEPs and of target-evoked potentials. Data were referred to the mean amplitude of the first 100 ms as baseline and were edited for artifacts, by rejecting trials with voltage differences≥150 µV, voltage steps≥30 µV, and voltages exceeding ±100 µV. By applying these criteria also to the EOG channels, segments with eye-blinks were rejected. Actually, eye-blinks rarely occur during the rapidly presented series.

As a further means of editing the data for artifacts, the averaged EOG waveforms contralateral minus ipsilateral to T1 were checked, from left and right hEOG in the x-axis block and from upper and lower vEOG in the y-axis block. Any participant's data were rejected if these averaged EOG difference waveforms deviated by 8 µV from baseline within 700 ms after T1 onset, indicating eye movements≥0.6° towards T1. As mentioned above in the “Participants” section, this criterion led to excluding 5 of the originally 23 participants.

Artifact-free segments were averaged over trials separately for each participant, and grand means over participants were formed for illustrating the results.

### Data analysis

For analysis of overt behavior, T1 identification rate was assessed as number of trials with correct T1 responses relative to all trials, and T2 identification rate was assessed as number of trials with correct T2 and T1 responses relative to all trials with correct T1 responses. These percentages of trials with correct responses were computed in each combination of T1 location, T1-T2 lag, and T2 location, separately for the x-axis and the y-axis block.

Segments for filler-VEP analysis spanned 1000 ms, from 100 ms before onset of the first stimulus pair to 900 ms afterwards. These segments were averaged over trials separately for x- and y-axis blocks, irrespective of success in target identification and of locations and time-points of the following T1 and T2. The mean number of accepted trials was 320 (226–349). The average waveforms consisted of a series of VEPs, evoked in intervals of 130 ms by the series of stimuli. The parameter of most interest was the lag between right and left visual cortex. Conventional measurement consisted in determining the P1 and N1 peaks of the first VEP (which were large and unambiguously identifiable) as most positive peak 70–170 ms and most negative peak 110–250 ms after stimulation onset, at the twelve posterior sites PO7 & PO8, PO9 & PO10, P7 & P8, P9 & P10, PO3 & PO4, O1 & O2, and entering their latencies and amplitudes to ANOVAs. A second method took the entire length of the waveform into account. The lag was determined by shifting each participant's waveforms of 800 ms duration from left sites against their symmetric right sites in 2 ms steps within ±50 ms, and selecting the shift that rendered the largest cross-correlation [26]. The duration of 800 ms was chosen to include the first five P1 and N1 peaks, ending before the N1 peak of the sixth stimulus pair which could already contain the T1 target. Latency shifts were tested against zero and between x-axis and y-axis blocks by t-tests.

Segments for T1 analysis ranged from 100 ms before to 700 ms after T1 onset and were averaged for trials with correct responses to both T1 and T2, separately for the four T1 locations (left, right, top, bottom). The mean number of accepted trials was 110 (56–167). To assess PCN in x-axis blocks and its possible correlate in y-axis blocks, mean amplitudes 200–220 ms measured at PO7 and PO8 were entered to ANOVA.

Segments for T2 analysis likewise started 100 ms before T1 and lasted until 500 ms after T2, i.e., for 730 ms with lag 1 and for 1120 ms with lag 4. Segments were averaged for trials with correct responses to both T1 and T2, separately for the four T2 locations (left, right, top, bottom), the two T1-T2 relations (same stream, stream change), and the two T1–T2 lags (1, 4). The mean number of accepted trials was 35 (23–45) for T2 after same-stream T1 at lag 1 and 26 (8–44) both after same- and different-stream T1 at lag 4. Numbers were lowest for T2 after other-stream T1 at lag 1, 22 (2–42). Thus, these data were affected by noise. Nevertheless, it was decided to use the full design for analysis. (By increasing the noise variance relative to the interesting variance, the low number of trials was expected to produce a conservative bias, if anything, in statistical testing). To distill the T2-evoked waveform from the lag 1 averages, independent of overlapping T1-evoked potentials, the first 730 ms of the lag 4 waveforms (averaged over same-stream and stream-change T2) including T1-evoked and background-evoked activity only, were subtracted from the lag 1 averages, and the mean of the 100 ms preceding T2 onset was subtracted as baseline from the data. With lag 4 waveforms, where T1 had preceded by 520 ms, the overlap issue reduced to slow waves evoked by T1. To cope with this, the mean of 200 ms preceding T2 onset was subtracted as baseline from the data. To assess T2-evoked PCN in x-axis blocks and its possible correlate in y-axis blocks, mean amplitudes were measured between 180 ms and 280 ms after T2 onset at PO7 and PO8 and entered to analysis.

ANOVA designs will be detailed in the Results section. Degrees of freedom were corrected by Greenhouse-Geisser's ε when repeated-measurement factors had three or more levels. Corrected p-values will be reported, but ε values will not be indicated, for brevity. Likewise, partial eta-squared will not be explicitly indicated, being easily derived from the reported F-values by the formula η_p_^2^ = (F/15)/(1+F/15). IBM SPSS statistics 20 was used. When interactions were significant, their sources were clarified by ANOVAs or t-tests on subsets of the data.

## Results

### Target identification

**Figure 2:**
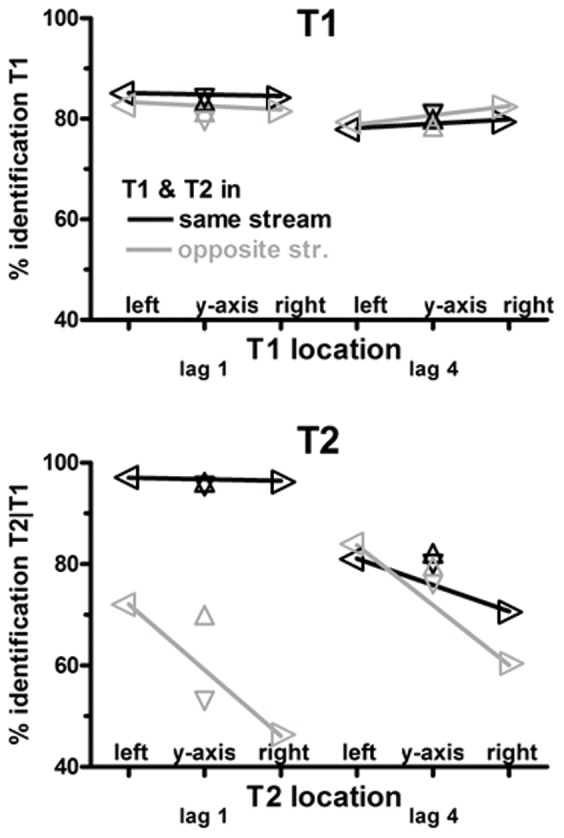
Rates of target identification. Percentages of correctly identified T1 (relative to all trials) are depicted in the upper panel, and of correctly identified T2 (in T1-correct trials, relative to all correct-T1 trials) in the lower panel. Values connected by lines denote values from the x-axis block. Black color denotes targets where the other target was in the same stream, grey color denotes targets where the other target was in the other stream. Left, right, upward and downward pointing triangles denote left, right, up, and down location, respectively.

Target-identification rates are compiled in Table 1 and are displayed in Figure 2. ANOVA on these rates had the factors Target Location (left, right, top, bottom; with "target" denoting T1 or T2, depending on analysis), T1–T2 Lag (1 vs. 4), and T1–T2 Stream Change (T1 and T2 in same vs. in opposite stream).

**Table 1:** Percentages of trials in which targets were correctly identified.

	streams on		x-axis		streams on		y-axis	
T1-T2 Lag	1		4		1		4	
target location	left	right	left	right	up	down	up	down
T1&T2 location					**T1**			
same	**85** (12)	**85** (13)	**78** (21)	**80** (15)	**83** (12)	**84** (12)	**80** (17)	**81** (17)
different	**83** (13)	**82** (12)	**79** (15)	**83** (12)	**81** (14)	**80** (14)	**78** (16)	**81** (12)
					**T2**			
same	**97** (3)	**96** (5)	**81** (16)	**71** (17)	**96** (5)	**95** (4)	**82** (20)	**80** (15)
different	**72** (19)	**46** (19)	**84** (16)	**60** (21)	**81** (16)	**70** (17)	**80** (19)	**76** (17)

Values are mean percentages across participants (SD). T1 percentages were computed as numbers of trials with correctly identified T1 relative to all trials. T2 percentages were computed as numbers of trials with correctly identified T1 and T2 relative to all correct-T1 trials.

#### T1

Responses were correct in 82% of trials. T1 was better identified when T2 followed at lag 1 than at lag 4, *F*_1,15_ = 10.5, *p* = 0.006. Other effects were not significant, in particular there was no effect of T1 Location (*F*_3,45_≤1.8, p≥0.17).

#### T2

T2 was better identified in the same stream as T1 than in the other, *F*_1,15_ = 139.1, p<0.001,. Interacting with T1-T2 lag, *F*_1,15_ = 105.3, p<0.001, this effect was very large with lag 1, such that T2 was identified nearly perfectly when following T1 in the same stream, and was much smaller with lag 4, though still significant (*F*_1,15_ = 15.3, p = 0.001 in separate analysis).

Of most interest, T2 Location had its effect, *F*_3,45_ = 12.8, p<0.001, interacting with Stream Change, *F*_3,45_ = 14.8, p<0.001, with Lag, *F*_3,45_ = 3.6, p = 0.03, and with Stream Change×Lag, *F*_3,45_ = 5.0, p = 0.005. To resolve these effects, the four T2 locations were compared to each other by means of t-tests for each of the four combinations of Stream Change×Lag. (We note that these t-tests and their p-values do not provide valid statistical inference but are meant as post-hoc test to clarify the reported main and interaction effects of Location.)

T2 in same stream as T1, lag 1: T2 was identified equally well at each position, t_15_<0.6, n.s.

T2 in opposite stream from T1, lag 1: T2 was better identified left than right, t_15_ = 6.0, p<0.001, and better above than below, t_15_ = 4.4, p<0.001. T2 identification did not differ between left and above, t_15_ = 0.5, n.s., nor between right and below, t_15_ = −1.0, n.s. In consequence, T2 was worse identified below than left, t_15_ = −4.1, p = 0.001, and better above than right, t_15_ = 4.5, p<0.001.

T2 in same stream as T1, lag 4: Identification rates did not differ among left, above, and below, t_15_≤0.7, n.s., but right T2 was worse identified than at the other locations: left, t_15_ = −4.0, p = 0.001; above, t_15_ = −2.6, p = 0.02; below, t_15_ = −2.2, p = 0.04.

T2 in opposite stream from T1, lag 4: T2 was better identified left than right, t_15_ = 6.6, p<0.001. Above and below did not differ, t_15_ = 0.9, n.s. Bottom T2 was worse identified than left T2, t_15_ = −2.3, p = 0.04, and better than right T2, t_15_ = 3.5, p = 0.003. Top T2 did not differ from left T2, t_15_ = −1.1, n.s., and was better identified than right T2, t_15_ = 4.2, p = 0.001.

To summarize:

1. T2 was better identified left than right (except for same-stream lag 1, due to the ceiling effect).
2. Midline T2 was never better identified than left T2. When T1 had been in the other stream, midline-bottom T2 was even worse identified than left T2.
3. Midline T2 was generally better identified than right T2 (except for same-stream lag 1, due to the ceiling effect, and for bottom T2 with different-stream lag 1).
4. Top and bottom T2 were identified equally well, except with different-stream lag 1 where T2 was much worse identified at bottom.

### Visual potentials evoked by the series of filler stimuli

**Figure 3:**
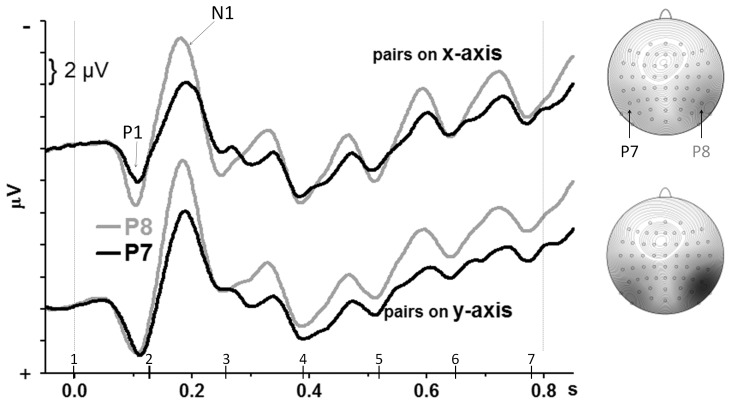
Potentials evoked during the first 0.85 s of the stimulus stream. The waveforms are grand means across participants recorded from P8 and P7 (right and left above visual cortex), starting at onset of the first pair of the stimulus series. Negative voltage points upwards. Numbered vertical bars at the x-axis denote the stimulus time-points. The dashed vertical lines at 0 ms and 800 ms denote the epoch for which the cross-correlation was computed for P8. The two heads on the right side denote the scalp topography of the first N1 (at 190 ms), showing largest amplitudes at P8 and PO7 (see Figure 4 for position of PO7 on the scalp). The scale spans from −10 µV (dark grey) to 0 µV (white). View on the head is from above.

Grand-average waveforms of the first 850 ms of each trial are displayed in Figure 3. The large P1 and N1 peaks evoked by the first pair of stimuli were measured at six posterior sites on either hemisphere. ANOVAs on amplitudes and latencies had the factors Electrode Pair (O1 & O2, PO3 & PO4, PO7 & PO8, PO9 & PO10, P7 & P8, P9 & P10), Hemisphere (left vs. right electrode site), and Axis of Stimulation (x vs. y axis). Main effects of Electrode Pair will not be reported in detail, for brevity.

There were no effects on P1 amplitude (except for a main effect of Electrode Pair). P1 latencies were earliest at P9&10 and latest at PO3&4 with x-axis stimuli, and were the same at all electrode pairs with y-axis stimuli (Axis×Electrode Pair: *F*_5,75_ = 3.2, p = 0.04).

N1 amplitudes were larger at right than left sites (Hemisphere: *F*_1,15_ = 7.0, p = 0.02) in the PO7&8, P7&8, and P9&10 pairs (Hemisphere×Electrode Pair: *F*_5,75_ = 8.4, p<0.001). Additionally, N1 was larger for stimuli on the y-axis than on the x-axis, i.e., for midline than for lateral stimuli (Axis: *F*_1,15_ = 14.3, p = 0.002). This effect did not interact with Hemisphere and Electrode Pair, F<0.9, n.s.

N1 latencies were earlier at right than left sites (Hemisphere: *F*_1,15_ = 5.3, p = 0.04). There were no effects of Axis and no interactions with Axis or Electrode Site.

To assess the latency difference between hemispheres by integrating more data than just the first N1 peak, the maximum cross-correlation was determined for different lags between waveforms from left and right sites. The P7&P8 pair was selected because N1 amplitudes were numerically largest (cf. maps in Figure 3) and because cross-correlations were slightly larger and less variable at this pair than at PO7&8 and PO3&4. An 800 ms epoch at P8, starting at onset of the first stimulus pair, was correlated to an 800 ms epoch for P7 which was defined to start at different time-points, from −50 ms (leftmost value of Figure 3) to +50 ms (ending at the rightmost value of Fig. 3). Confirming results of N1 peak latency, waveforms at the right hemisphere (P8) were slightly leading before waveforms at the left hemisphere (P7), by 5.1 ms (±9 ms SD) with x-axis stimuli and by 4.6 ms (±8 ms SD) with y-axis stimuli. These lags were larger than zero (t_15_ = 2.35 and 2.25 for x-axis and y-axis, p = 0.03 and p = 0.04) and did not differ from each other (t_15_ = 0.3, n.s.).

### T1-evoked potentials

**Figure 4:**
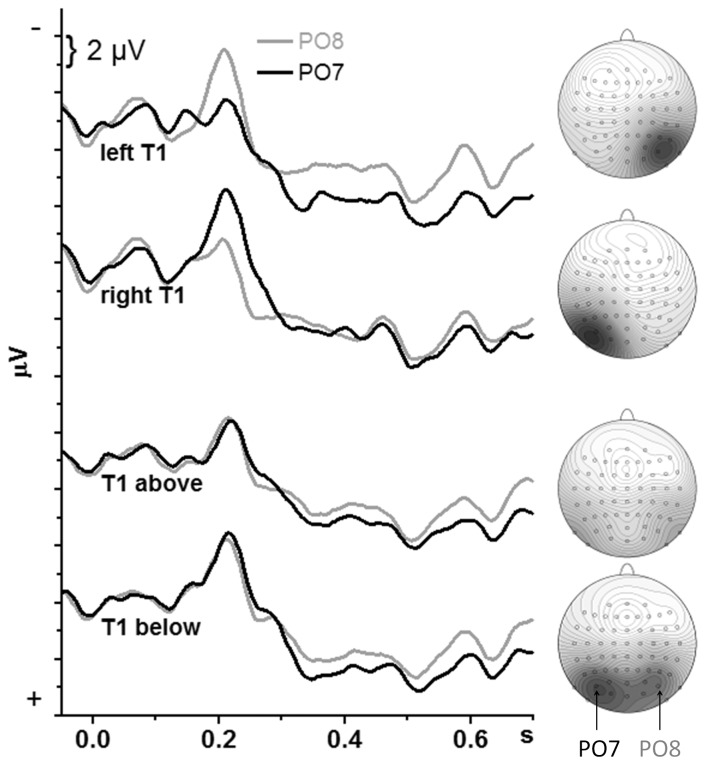
Potentials evoked by T1. The waveforms are grand means across participants recorded from PO8 and PO7 (right and left above visual cortex). Time-point zero is T1 onset. Negative voltage points upwards. The upper two panels depict data from the x-axis block, the lower two panels from the y-axis block. The maps on the right denote the scalp topography at the negative peak around 220 ms. The scale is −6 µV (dark grey) to +6 µV (white). View on the head is from above.

Grand-average waveforms of the T1-evoked potentials are displayed in Figure 4. The PO7 & PO8 pair of recording sites is displayed and will be analyzed because PCN amplitudes were largest at these sites (cf. maps in Figure 4). Mean amplitudes 200–220 ms were entered to analysis. First, an overall ANOVA was conducted, with the factors T1 Location (left, right, top, bottom) and Hemisphere (left vs. right, i.e., PO7 vs. PO8). (The factors Stream Change and Lag from T1 to T2 did not apply because the T1-evoked peak at 200 ms was not expected to be affected by the following T2). The effect of T1 Location, *F*_3,45_ = 4.4, p = 0.01, was resolved in separate ANOVAs for all pairs of T1 Location which showed that largest amplitudes were evoked by bottom T1 (*F*_1,15_≥5.2, p≤0.04) while left, right, and above did not differ from each other (*F*_1,15_≤1.1, n.s.). The main effect of Hemisphere was not significant, *F*_3,45_ = 0.0, but its interaction with T1 Location was, *F*_3,45_ = 50.5, p<0.001, prompting separate ANOVAs for x- and y-axis, with the factors T1 Location (left vs. right for x-axis, top vs. bottom for y-axis) and Hemisphere.

For stimuli in the x-axis, the only effect was a strong interaction of T1 Location×Hemisphere, *F*_1,15_ = 66.3, p<0.001, because, as could be expected, N1 was larger contralaterally than ipsilaterally to T1, reflecting the PCN effect. In detail, N1 was larger at PO8 than PO7 with left T1, t_15_ = 5.8, p<.001, and larger at PO7 than PO8 with right T1, t_15_ = 5.0, p<.001.

For stimuli in the y-axis, larger N1 amplitudes were evoked by bottom than top T1 (T1 Location: *F*_1,15_ = 14.0, p = 0.002). Of interest, there was a difference between hemispheres in this effect, T1 Location×Hemisphere *F*_1,15_ = 4.9, p = 0.04, because the increase from top to bottom T1 was larger at PO7 (t_15_ = 4.4, p = 0.001) than at PO8 (t_15_ = 2.8, p = 0.01). This interaction could not be resolved to differences between PO7 and PO8 with either top T1, t_15_ = 0.8, n.s., or bottom T1, t_15_ =  −0.5, n.s., though.

### T2-evoked potentials

**Figure 5:**
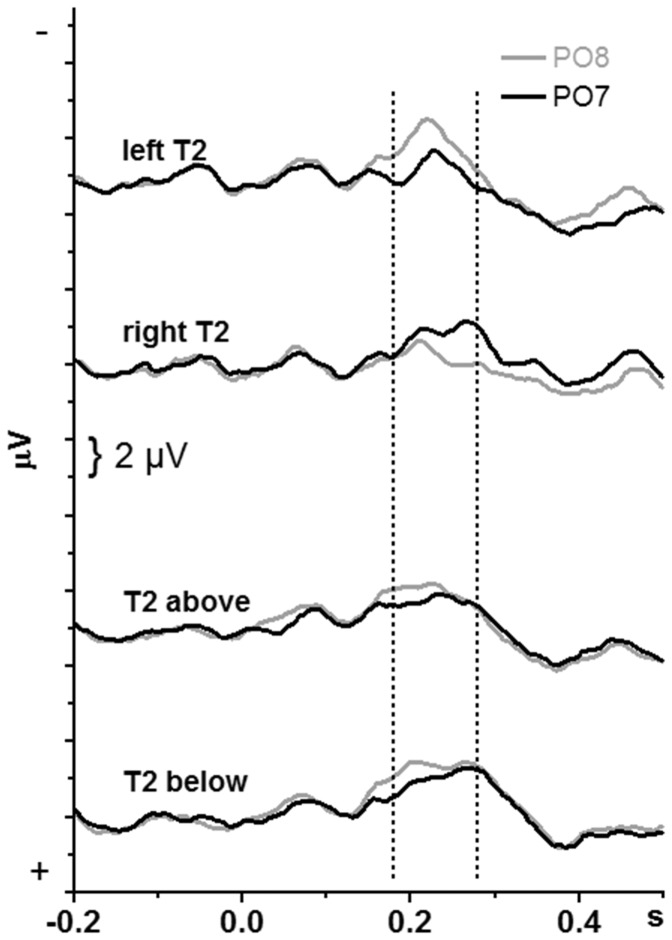
Potentials evoked by T2, pooled across T1–T2 relations. The waveforms are grand means across participants recorded from PO8 and PO7 (right and left above visual cortex). Time-point zero is T2 onset. Negative voltage points upwards. The upper two panels depict data from the x-axis block, the lower two panels from the y-axis block. These data have been averaged from the four T1–T2 relations separately illustrated in Figure 6. The dotted vertical lines denote the analyzed interval.

**Figure 6:**
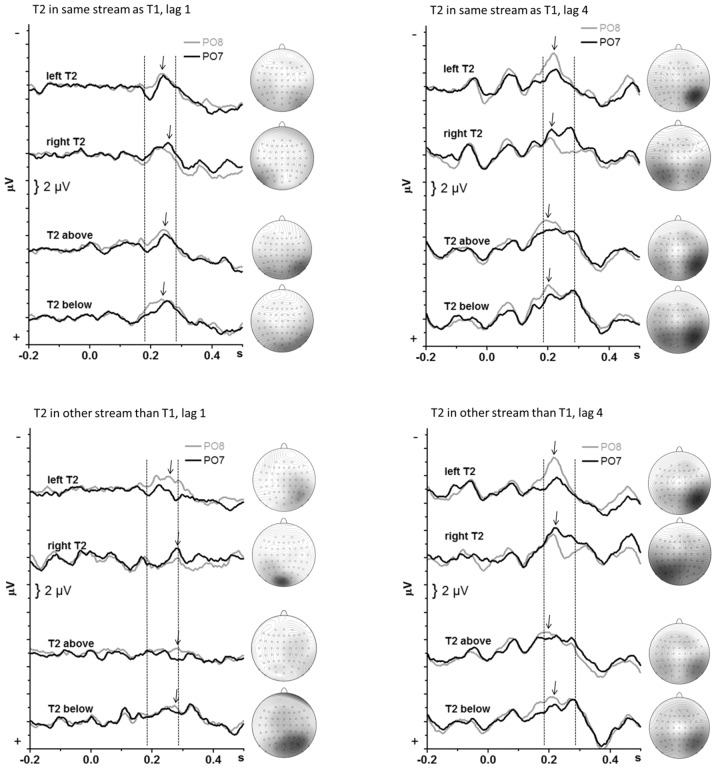
Potentials evoked by T2, separately for each T1–T2 relation. The waveforms are the same as in Figure 6 but are displayed separately for each of the four T1–T2 relations. The dotted vertical lines denote the analyzed interval. The maps on the right denote the scalp topography at the negative peak, with the mapped time-point denoted by the arrows in the waveforms. The scale of the maps is −4 µV (dark grey) to +4 µV (white). View on the head is from above.

Grand-average waveforms of the T2-evoked potentials are displayed in Figures 5 and 6 for the PO7 & PO8 pair of sites. Figure 5 displays data pooled across the four combinations of stream change and lag, to present the data concisely, comparably to the T1-evoked potentials in Figure 4. Figure 6 shows each of the four combinations of stream change and lag separately. Because the T2-evoked N1 and PCN were not as distinct as with T1, mean amplitudes were measured in a wider interval of 100 ms, between 180 ms and 280 ms after T2 onset (marked in Figures 5 and 6 by the vertical dotted lines) with this time-range split in two 50 ms epochs. The overall ANOVA on this measure had five factors. These were Hemisphere (PO7 vs. PO8) and Epoch (182–230 ms, 232–280 ms) plus the three factors used for analysis of identification rates: T2 Location (left, right, top, bottom), T1–T2 Lag (1 vs. 4), and T1–T2 Stream Change (same stream vs. opposite stream).

Of most interest were effects of Hemisphere. As indicated by the Epoch × Hemisphere interaction, *F*_1,15_ = 23.8, p<0.001, amplitudes were larger at PO8 than PO7 in the 182–230 ms epoch (separate effect of Hemisphere: *F*_1,15_ = 8.8, p = 0.01) and did not differ in the 232–280 ms epoch (*F*_1,15_ = 0.1, n.s.). Of importance, this effect occurred equally for both axes: When separate ANOVAs were computed for x- and y-axis, amplitudes were larger at PO8 than PO7 in the 182–230 ms epoch both with x-axis stimuli (separate effect of Hemisphere in this epoch: *F*_1,15_ = 7.3, p = 0.02) and with y-axis stimuli (Hemisphere: *F*_1,15_ = 8.8, p = 0.009). This Epoch×Hemisphere interaction was modified by Lag (*F*_1,15_ = 13.3, p = 0.002), by T1–T2 Stream Change (*F*_1,15_ = 7.6, p = 0.02), and by Lag×Stream Change (*F*_1,15_ = 14.0, p = 0.002). Therefore, Hemisphere effects were computed separately for either epoch in each of the four combinations of Lag×Stream Change displayed in Figure 6. For three of four Lag×Stream Change combinations (except Lag 1 with side change from T1), amplitudes were larger at PO8 than PO7 in the first epoch (*F*_1,15_≥5.1, p≤0.04) and did not differ in the second epoch. For Lag 1 with side change from T1, amplitudes were larger at PO8 than at PO7 in the second epoch only (*F*_1,15_ = 7.7, p = 0.01), which reflected the delay of waveforms in this condition.

The interaction of Hemisphere with T2 Location, *F*_3,45_ = 22.3, p<0.001, reflected the expected PCN with x-axis stimuli, i.e., enhanced negativity contralateral to T2: Hemisphere×T2 Location with x-axis *F*_1,15_ = 27.8, p<0.001; with y-axis *F*_1,15_ = 0.8, n.s. This PCN differed between lags, T2 Location×Hemisphere×Lag, *F*_3,45_ = 5.7, p = 0.006 (T2 Location×Hemisphere×Lag for x-axis stimuli: *F*_1,15_ = 9.3, p = 0.008; for y-axis stimuli: *F*_1,15_ = 2.8, n.s.), by being more distinct with lag 4 (T2 Location×Hemisphere at x-axis, lag 4: *F*_1,15_ = 31.7, p<0.001) than with lag 1 (lag 1: *F*_1,15_ = 11.0, p = 0.005).

Independently of Hemisphere, the main effect of T2 Location, *F*_3,45_ = 6.4, p = 0.004, reflected larger negative amplitudes of T2 below than left, right, and above (in separate pair-wise analyses *F*_1,15_≥6.7, p≤0.02) which did not differ from each other (*F*_1,15_≤3.3, p≥0.09). Further, amplitudes were larger with lag 4 than with lag 1, *F*_1,15_ = 12.1, p = 0.003, particularly but not only in the earlier epoch (Lag×Epoch: *F*_1,15_ = 19.9, p<0.001) and when T1 had been on the same side, *F*_1,15_ = 4.6, p = 0.049.

## Discussion

In order to find clues about which hemisphere preferentially processes filler stimuli and targets presented at vertical midline in rapid serial visual presentation and about whether such stimuli are processed in a cooperative manner, better than either hemisphere could do alone, EEG potentials and target identification were compared between streams presented above and below fixation to streams presented left and right from fixation.

### VEPs evoked by filler stimuli

From the N1 peak evoked by the first filler-letter frame onwards, there was a small reliable latency advantage for right over left-hemisphere waveforms. Likewise, N1 amplitudes (at least for the first stimulus where they could be unequivocally measured) were considerably larger at right than left recording sites. These results suggest that the presented symmetrical frames were processed earlier and induced more activation in the right than in the left hemisphere, which seems to be specific to the present task (cf. Discussion in [18]). These latency and amplitude advantages of the right hemisphere were equally present for top-and-bottom streams as for left-and-right streams, suggesting preferential processing of these dual streams in the right hemisphere both when presented at vertical midline and at horizontal midline.

N1 was generally larger for vertical than for lateral pairs. We suspect that this difference was driven by the lower stimulus of the vertical pair, because also lower targets (T1 and T2) evoked larger negative amplitudes than targets above, left, or right, whereas targets above did not differ from left or right. This effect was probably simply due to the projection of the lower visual field to visual areas nearer to the upper cortical surface, thus nearer to the recording electrodes [27], [28].

### T1: Identification rates and EEG asymmetry

Relevant effects on rates of T1 identification were absent, T1 being equally well identified left, right, above and below fixation. This might be due to a ceiling effect. But on the other hand, the level of 82% identification rate is still well below 100%, and T1 identification did improve beyond such levels in previous studies, when stimuli acted as temporal cues in the stream before T1 [29] or when T1 was embedded in simpler distractors than letters (unpublished data from our lab). Thus, improvements of T1 identification over the rates obtained with lateral stimuli do not appear impossible. No such improvements were obtained, which speaks against the notion of cooperation.

As could be expected, contralateral negativity was evoked by laterally presented T1 (cf. [15], [18]) to the same extent for left and right T1. Thus, no hemispheric preference was seen: T1 simply was processed in the contralateral hemisphere. Similarly, no distinct hemispheric preference was seen with vertical-midline T1. This might be considered trivial if it were not for the asymmetries in the preceding VEPs and in the following T2-evoked negativity. Thus, the lack of midline-T1-evoked asymmetry needs some explanation. Actually, there even was a weak tendency, reflected by the significant Hemisphere×T1 Location interaction with y-axis stimuli, for preferred left-hemisphere activation, particularly with bottom T1. It may be assumed that the usual right-hemisphere preference was not obtained, both with lateral and midline T1, because T1 identification was easy (due to its unique color and the restricted target set) such that the left hemisphere could cope with this task as well. Alternatively, the balanced T1-evoked activation might be due to "breathtaking" by the right hemisphere (cf. [30]): After having preferentially processed the filler stimuli, or due to its facing the difficult task of identifying T2, the right hemisphere may lose its dominance and leave some room for the left hemisphere to win the interhemispheric race.

### T2 identification rates: Lacking redundancy gain

Targets presented at midline might be processed by the left or the right hemisphere or by both. As argued in the Introduction, if both hemispheres cooperate, the result might be better than the result achieved by either hemisphere alone. T2 was reliably better identified on the left than on the right, as in previous studies [13]–[18]. Yet midline T2, be it above or below fixation, was never better identified than left T2. Thus, evidence for cooperation was absent. Rather, the most parsimonious interpretation, in accordance with the racing model, is that these midline targets were preferentially processed by the right hemisphere.

This lacking redundancy gain may appear in conflict with studies that did show gains of involving both hemispheres. Yet, many of those studies presented two distinct stimuli, one in either hemifield (e.g., [14], [31]–[33]) whereas the present study deals with one target stimulus at vertical midline. The results might be seen in conflict with results obtained by Hunter et al. [34], though. In that study, single words were presented in the usual format, with letters from left to right. Words were named fastest when crossing fixation, such that the smaller part of the word was in the left and the larger part in the right hemifield. Because this was faster than when words were entirely in the right hemifield, Hunter et al. [34] concluded that hemispheric cooperation is beneficial for word reading. Yet, other factors might have played a role in that study. As was suggested by Marc Brysbaert, coauthor of [34], as a reviewer of the present manuscript, the apparent divergence may be explained by benefits of better perceptual resolution when words crossed fixation in that study because then all letters were necessarily presented nearer to the fovea (zone of best perceptual resolution) [35]. We conclude that the lack of redundancy gain for our target letters at vertical midline fits the preceding literature.

### T2 identification above versus below fixation

Top and bottom T2 generally were equally well identified except with different-stream lag 1 where bottom T2 was much worse identified than top T2.

It is not a matter of course that top and bottom T2 generally did not differ. Because the upper visual field may have more direct access to the ventral pathway [36] supposed to be more specialized in object identification than the dorsal pathway [37]. The specific deficit in identifying bottom T2 when it very quickly followed top T1 might be due to this putative general disadvantage of the lower visual field. It might have needed this most difficult condition to bring out this structural disadvantage. Alternatively, this disadvantage might reflect differences in how quickly attention is moved down from top compared to up from bottom, evident in this most difficult condition only. Unfortunately, we are not aware of previous published evidence on such differences. Alternatively, this disadvantage might relate to left-right asymmetries. There is evidence that, at least with short presentation times as used in our study, stimuli induce a stronger leftward perceptual bias (indicative of right-hemisphere processing) in the upper than in the lower visual field [38]. Thus, T1 presented at top would be preferentially processed by the right hemisphere. Consequently, with T1 still being processed, the left hemisphere might have had more capacity to process T2 which, by quickly following at a different location, could not be processed in the same episode as T1 (cf. [39]). As argued in the Introduction, such advantage in processing may imply inhibiting the other hemisphere. Therefore, bottom T2 in this particular sequence would be processed by the left hemisphere and, therefore, identified at the same poorer quality as right T2.

### T2-evoked negativity

In contrast to T1, T2-evoked negativity was asymmetric, both with lateral and midline streams. With lateral streams, contralateral right-hemisphere negativity evoked by left T2 occurred earlier than contralateral left-hemisphere negativity evoked by right T2. This result replicated earlier findings [18]. Of interest, the same applied to midline streams: Right-hemisphere negativity was preponderant in the early 180–230 ms epoch. This is evidence that midline-T2 was preferentially processed in the right hemisphere, supporting and extending the conclusions drawn above from identification rates.

One interpretation proposed above for the poor identification of bottom T2 after top T1 preceding at lag 1 referred to a putative closer link of the lower visual field to the left hemisphere. This interpretation implies that a left-hemisphere negative focus should be obtained in this particular condition. This was not the case, though (cf. lower left panel of Figure 6). The alternative interpretations of less direct links from the lower visual field to the ventral pathway or of faster bottom-up than top-down shifts of attention lead to the prediction that T2-evoked negativity should be delayed in this particular condition relative to the opposite case (T2 above, at lag 1 after T1 below). This result was not obtained either. Rather, T2-evoked negativity was generally delayed with stream change from T1 to T2 at lag 1 relative to the other conditions (cf. lower left panel of Figure 6 relative to the three other panels). Thus, the precise mechanisms for this special case remained elusive. One reason for this failure may be the poor signal/noise ratio of EEG in this particular condition because not many trials remained for analysis in this condition due to the low rate of target identification.

### Principal and methodological considerations on processing of midline stimuli

We had started from the assumption that both visual cortices get activated by midline stimuli. From there we proceeded to ask which hemisphere will further preferentially process the signals producing this activation, and whether there is racing or cooperation in this processing. However, the nature of the first bilateral activation is far from clear. For example, when an "L" is presented at midline, will then its left part (the "I") be transmitted to the right hemisphere, and the right part (the "_") to the left? If so, where will these two elements be integrated? The problem is not solved by assuming that the fovea projects to both hemispheres (e.g., [4], [5]) because midline stimuli may lie outside the zone of foveal vision as well, above and below fixation. Thus, an early stage of interhemispheric integration has to be postulated, not needed for lateral stimuli. Alternatively, the fact may be emphasized that square angles and straight separations are the exception rather than the rule in physiological systems, such that under normal conditions the object ("L") may well reach one of the two hemispheres in its entirety. For example, participants might have kept their fixation slightly off physical midline, e.g., at the right tip of the fixation cross, 0.1° right of fixation, which is too small to be noticed by our eye-tracker. Thereby, stimuli will be slightly shifted towards the left visual field and have greater chances of directly accessing the right hemisphere. Moreover, even if there was perfect straight-ahead fixation and even if there are sharp boundaries between the retinal cells sensitive to the left or right hemi-fields, it is improbable that these boundaries will follow a straight line as drawn by a ruler. Thus, it is difficult if not impossible to exclude any anatomical asymmetry that will give advantage to one hemisphere even when participants keep perfect fixation. Rather, such asymmetry may be the normal case when objects are visually perceived at vertical midline.

## Conclusion

This study used behavioral and neurophysiological markers of lateralized processing to investigate hemisphere-specific processing of stimuli presented at vertical midline. We arrived at the conclusion that the right-hemisphere dominance obtained in this task with left and right stimuli also applies to these stimuli when presented at midline. The asymmetries of VEPs evoked by the filler stimuli, of T1-evoked negativity, and of T2-evoked negativity, suggest that the default setting for perceiving the series of filler letters at vertical midline is by giving preference to the right hemisphere, that there is no such tendency for T1, and that then T2 is preferably processed in the right hemisphere again. Identification rates both of T1 and T2 provided no evidence for cooperation between hemispheres in identifying stimuli presented at vertical midline, rather supporting the assumption of competitive race.

## References

[1] Leff A (2004) A historical review of the representation of the visual field in primary visual cortex with special reference to the neural mechanisms underlying macular sparing. Brain and Language 88: 268–278. 14967211 10.1016/S0093-934X(03)00161-5

[2] Zeki S (1993) A vision of the brain. Oxford (GB): Blackwell Scientific Publications.

[3] Ellis AW, Brysbaert M (2010) Split fovea theory and the role of the two cerebral hemispheres in reading: A review of the evidence. Neuropsychologia 48: 353–365. 19720073 10.1016/j.neuropsychologia.2009.08.021

[4] Jordan TR, Paterson KB (2009) Re-evaluating split-fovea processing in word recognition: A critical assessment of recent research. Neuropsychologia 47: 2341–2353. 18723038 10.1016/j.neuropsychologia.2008.07.020

[5] Marzi CA, Mancini F, Sperandio I, Savazzi S (2009) Evidence of midline retinal nasotemporal overlap in healthy humans: A model for foveal sparing in hemianopia? Neuropsychologia 47: 3007–3011. 19465034 10.1016/j.neuropsychologia.2009.05.007

[6] Putnam MC, Steven MS, Doron KW, Riggall AC, Gazzaniga MS (2010) Cortical projection topography of the human splenium: Hemispheric asymmetry and individual differences. J Cogn Neurosci 22: 1662–1669. 19583478 10.1162/jocn.2009.21290

[7] Gazzaniga MS (2005) Forty-five years of split-brain research and still going strong. Nature Rev Neurosci 6: 653–659. 16062172 10.1038/nrn1723

[8] Greene DJ, Zaidel E (2011) Hemispheric differences in attentional orienting by social cues. Neuropsychologia 49: 61–68. 21093465 10.1016/j.neuropsychologia.2010.11.007

[9] Verleger R, Binkofski F, Friedrich M, Sedlmeier P, Kömpf D (2011a) Anarchic-hand syndrome: ERP reflections of lost control over the right hemisphere. Brain & Cognition 77: 138–150. 21703748 10.1016/j.bandc.2011.05.004

[10] Rossion B, Joyce CA, Cottrell GW, Tarr MJ (2003) Early lateralization and orientation tuning for face, word, and object processing in the visual cortex. NeuroImage 20: 1609–1624. 14642472 10.1016/j.neuroimage.2003.07.010

[11] Miller J (1982) Divided attention: Evidence for coactivation with redundant signals. Cogn Psychol 14: 247–279. 7083803 10.1016/0010-0285(82)90010-x

[12] Kinsbourne M (1987) Mechanisms of unilateral neglect. In Jeannerod M, editor. Neurophysiological and neuropsychological aspects of spatial neglect. Amsterdam (NL): Elsevier. p 69–86.

[13] Holländer A, Corballis MC, Hamm JP (2005) Visual-field asymmetry in dual-stream RSVP. Neuropsychologia 43: 35–40. 15488903 10.1016/j.neuropsychologia.2004.06.006

[14] Scalf PE, Banich MT, Kramer AF, Narechania K, Simon CD (2007) Double take: Parallel processing by the cerebral hemispheres reduces the attentional blink. J Exp Psychol: Hum Percept Perform 33: 298–329. 17469970 10.1037/0096-1523.33.2.298

[15] Verleger R, Sprenger A, Gebauer S, Fritzmannova M, Friedrich M, et al (2009) On why left events are the right ones: Neural mechanisms underlying the left-hemifield advantage in rapid serial visual presentation. J Cogn Neurosci 21: 474–488. 18564053 10.1162/jocn.2009.21038

[16] Śmigasiewicz K, Shalgi S, Hsieh S, Möller F, Jaffe S, et al (2010) Left visual-field advantage in the dual-stream RSVP task and reading direction: A study in three nations. Neuropsychologia 48: 2852–2860. 20546763 10.1016/j.neuropsychologia.2010.05.027

[17] Verleger R, Möller F, Kuniecki M, Śmigasiewicz K, Groppa S, et al (2010) The left visual-field advantage in rapid visual presentation is amplified rather than reduced by posterior-parietal rTMS. Exp Brain Res 203: 355–365. 20401472 10.1007/s00221-010-2237-z

[18] Verleger R, Śmigasiewicz K, Möller F (2011b) Mechanisms underlying the left visual-field advantage in the dual stream RSVP task: Evidence from N2pc, P3, and distractor-evoked VEPs. Psychophysiology 48: 1096–1106. 21265863 10.1111/j.1469-8986.2011.01176.x

[19] Luck SJ, Fan S, Hillyard SA (1993) Attention-related modulation of sensory-evoked brain activity in a visual search task. J Cogn Neurosci 5: 188–195. 23972153 10.1162/jocn.1993.5.2.188

[20] Wascher E, Wauschkuhn B (1996) The interaction of stimulus- and response-related processes measured by event-related lateralisations of the EEG. Electroencephalogr Clin Neurophysiol 99: 149–162. 8761051 10.1016/0013-4694(96)95602-3

[21] Verleger R, Żurawska vel Grajewska B, Jaśkowski P (2012a) Time-course of hemispheric preference for processing contralateral relevant shapes: P1pc, N1pc, N2pc, N3pc. Adv Cogn Psychol 8: 19–28. 22419963 10.2478/v10053-008-0098-9 PMC3303108

[22] Jakowski P, van der Lubbe RHJ, Schlotterbeck E, Verleger R (2002) Traces left on visual selective attention by stimuli that are not consciously identified. Psychol Sci 13: 48–54. 11894850 10.1111/1467-9280.00408

[23] Wascher E (2005) The timing of stimulus localization and the Simon effect: An ERP study. Exp Brain Res 163: 430–439. 15711792 10.1007/s00221-004-2198-1

[24] Jolicœur P, Sessa P, Dell'Acqua R, Robitaille N (2006) On the control of visual spatial attention: evidence from human electrophysiology. Psychol Research 70: 414–424. 10.1007/s00426-005-0008-4 16184394

[25] Kiefer M, Brendel D (2006) Attentional modulation of unconscious 'automatic' processes: Evidence from event-related potentials in a masked priming paradigm. J Cogn Neurosci 18: 184–198. 16494680 10.1162/089892906775783688

[26] Okon-Singer H, Podlipsky I, Siman-Tov T, Ben-Simon E, Zhdanov A, et al (2011) Spatio-temporal indications of sub-cortical involvement in leftward bias of spatial attention. NeuroImage 54: 3010–3020. 21056675 10.1016/j.neuroimage.2010.10.078

[27] Kelly SP, Gomez-Ramirez M, Foxe JJ (2008) Spatial attention modulates initial afferent activity in human primary visual cortex. Cereb Cortex 18: 2629–2636. 18321874 10.1093/cercor/bhn022 PMC2733320

[28] Luck SJ, Girelli M, McDermott MT, Ford MA (1997) Bridging the gap between monkey neurophysiology and human perception: An ambiguity resolution theory of visual selective attention. Cogn Psychol 33: 64–87. 9212722 10.1006/cogp.1997.0660

[29] Verleger R, Śmigasiewicz K, Michael L, Niedeggen M (2012b) Effects of premature lure stimuli on 2^nd^-target identification in rapid serial visual presentation: Inhibition induced by lures or by 1^st^ target? Psychophysiology 49: 1254–1265. 22823332 10.1111/j.1469-8986.2012.01408.x

[30] Hellige JB, Cox PJ, Litvac L (1979) Information processing in the cerebral hemispheres: Selective hemispheric activation and capacity limitations. J Exp Psychol: General 108: 251–279. 10.1037//0096-3445.108.2.251 528905

[31] Delvenne J-F, Holt JL (2012) Splitting attention across the two visual fields in visual short-term memory. Cognition 122: 258–263. 22113121 10.1016/j.cognition.2011.10.015

[32] Kraft A, Pape N, Hagendorf H, Schmidt S, Naito A, et al (2007) What determines sustained visual attention? The impact of distracter positions, task difficulty and visual fields compared. Brain Res 1133: 123–135. 17174284 10.1016/j.brainres.2006.11.043

[33] Miniussi C, Girelli M, Marzi CA (1998) Neural site of the redundant target effect: electrophysiological evidence. J Cogn Neurosci 10: 216–230. 9555108 10.1162/089892998562663

[34] Hunter ZR, Brysbaert M, Knecht S (2007) Foveal word reading requires interhemispheric communication. J Cogn Neurosci 19: 1373–1387. 17651009 10.1162/jocn.2007.19.8.1373

[35] Brysbaert M, Nazir T (2005) Visual constraints in written word recognition: evidence from the optimal viewing-position effect. J Res Reading 28: 216–228.

[36] Previc FH (1998) The neuropsychology of 3-D space. Psychol Bull 124: 123–164. 9747184 10.1037/0033-2909.124.2.123

[37] Milner AD, Goodale MA (1995) The visual brain in action. Oxford (GB): Oxford University Press.

[38] Thomas NA, Elias LJ (2011) Upper and lower visual field differences in perceptual asymmetries. Brain Res 1387: 108–115. 21362412 10.1016/j.brainres.2011.02.063

[39] Wyble B, Potter MC, Bowman H, Nieuwenstein M (2011) Attentional episodes in visual perception. J Exp Psychol: General 140: 488–505. 10.1037/a0023612 PMC3149751 21604913

